# Imbalance in obesity and mental health among “little emperors” in China

**DOI:** 10.1371/journal.pone.0207129

**Published:** 2019-04-10

**Authors:** Ziwen Peng, Zhongyan Zheng, Hongying Han, Chenjie Dong, Jingjing Liang, Jianping Lu, Zhen Wei

**Affiliations:** 1 Department of Child Psychiatry, Shenzhen Kangning Hospital, Shenzhen University School of Medicine, Shenzhen, China; 2 Center for Studies of Psychological Application, School of Psychology, South China Normal University, Guangzhou, China; 3 Guangdong Key Laboratory of Mental Health and Cognitive Science, South China Normal University, Guangzhou, China; 4 Department of Psychiatry, the Third Affiliated Hospital, Sun Yat-Sen University, Guangzhou, China; 5 Department of Child Psychiatry, the Affiliated Shenzhen Maternity & Child Healthcare Hospital, Southern Medical University, Shenzhen, China; Hong Kong Polytechnic University, HONG KONG

## Abstract

**Introduction:**

Previous research has indicated that only children (i.e., those living with no siblings) have higher odds of obesity during childhood and young adulthood, compared with those living with siblings. However, little is known about whether the developing difference in overweight/obesity is accompanied by a difference in mental health (i.e., internalizing symptoms of depression and anxiety).

**Methods:**

The subjects for this prospective study were a randomly generated cohort of 1348 high-school students in Guangzhou, China. Participants completed assessments of anthropometric indices, lipid profiles, family-based factors, lifestyle, and internalization of symptoms (including those of depression and anxiety).

**Results:**

Compared to their peers with siblings, only children (adjusted odds ratio [aOR] = 1.68, 95% confidence interval [CI] [1.06, 2.65]) had significantly higher risk for obesity. However, only children with overweight/obesity had lower OR for depression at follow-up (aOR = 0.19, 95% CI [0.34, 0.86]), compared to individuals who were overweight/obese with siblings. This relationship was not significant for non-overweight individuals. No significant relationship between the number of siblings and anxiety at follow-up was observed, regardless of body mass index (BMI).

**Conclusions:**

Although being an only child was significantly associated with overweight and obesity among adolescents in China, participants with history of overweight/obesity are less likely to experience symptoms of depression associated with being an only child.

## Introduction

Adolescent obesity is a major public health concern that has recently increased in prevalence [1, 2]. Among 7- to 18-yr-old boys and girls in China, the prevalence of overweight increased from 1.81% in 1985 to 24.2% in 2014. Among 7- to 18-yr-old boys and girls in China, the prevalence of obesity increased from 3.15% in 1985 to 14.6% in 2014 [3, 4]. A similar rising prevalence of overweight and obesity has been reported worldwide [5]. Total fertility rate has simultaneously declined in many countries [6, 7], with the proportion of one-child families increasing and sib-size decreasing. The Chinese government started promoting and implementing the one-child policy (OCP) in 1979 to ease the enormous pressure of population explosion [8, 9]. Only children born following the OCP are often called “little emperors” and are accused of being selfish, maladjusted, and spoiled [10]. According to critics, one source of the problem is the “four-two-one” family structure that has become widespread in China since implementation of the OCP. Such a family consists of four grandparents, two parents, and one child; the only child in the family is doted on by the parents and grandparents [11].

Although only children may be blessed with relatively more family and social resources, those who have no siblings for company may face physical and socio-psychological problems during development [6, 12]. Compared with children who have siblings, only children have elevated risk for overweight and obesity [13, 14], and are equally at risk for engaging in negative psychosocial consequences, such as increasing sense of loneliness, sadness, and nervousness [12]. Only children have historically been portrayed as having undesirable personalities and exhibiting selfish social behavior [10, 12]. This stereotype is common in developing countries with low fertility rates (e.g., China since the 1980s), where the public perception is that only children are socially inept, selfish, anxious, dependent, and generally maladjusted [10, 11, 15]. Given these risks, there is a need for research to better understand the developing differences in prevalence of overweight and obesity between only children and children with siblings. This study was performed to investigate whether the difference in prevalence of overweight/obesity is associated with differences in mental health. Studies show increased likelihood of overweight and obesity among adolescents living with no siblings. These adolescents may become overweight and obese partly through being exposed to obesogenic behaviors and environments at home [16]. For example, previous studies in the United States and Japan showed that only children had higher odds of overweight or obesity, compared with children who had one or more siblings [16, 17]. One recent study that included participants from 25 provinces in China showed that being an only child significantly decreases one’s subjective well-being and that more intensive exposure to the one-child policy makes individuals more depressed and less happy [18]. However, relatively little is known about whether sib-size (i.e., living without siblings or living with siblings) affects the relationship between mental health and overweight/obesity among adolescents in China. Adolescence is a vulnerable period for obesity. No study to date has compared only children and children with siblings as part of a cross-sectional investigation of the medium- to long-term mental health effects of obesity. Therefore, there is a critical need for studies that explore the potential association between mental health and overweight/obesity, as well as sib-size, in young adolescents.

To address this gap in knowledge, we sought to design a cohort study that included “community-based sample”. In other words, we followed a cohort of young people, including only children and those with siblings, who were free of anxiety and depression screened via a health survey at baseline but varied in terms of overweight/obesity status. Mental health outcomes were assessed at the end of the follow-up period. This prospective study sought to: (1) examine risk for overweight and obesity among children with siblings, compared to only children; (2) investigate how overweight/obesity status in children with siblings, compared with only children, affects mental health. Based on the results of prior studies, we hypothesized that only children would have an increased risk for overweight and obesity, compared to those with siblings. We also hypothesized that only children who were free of anxiety and depression but also exhibited overweight/obesity would have higher risk for anxiety and depression, compared with non-only children with overweight/obesity.

## Materials and methods

### Participants and procedures

This longitudinal health survey was conducted in Guangzhou city, Guangdong Province, southeast China. Guangdong Province is the most populous province in China; Guangzhou city is the capital of Guangdong Province. The investigation were based on two projects, one is titled as multidimensional assessment standards of sub-health in adolescents supported by the national high-tech research and development program (2006AA02Z427) and another project is named mental health in adolescents in Guangdong province supported by the Department of Psychological Education of Elementary and Secondary Schools of the Guangdong Province Administration. The participants were recruited from July 2008 to December 2009. We carry out the investigation according to the following steps. First, we signed informed consent with each participating school and the principal of each selected classroom. Then, the parents or guardians of the students were informed of the study through a written notice sent home from the schools asking them to contact the teachers by phone if they did not wish their child to participate in the survey before one week on screening day. Next, the team members explained the anonymous and confidential nature of the data to the students, and provided an opportunity for them to ask questions prior to the formal investigation. If they were not willing to participate, they were allowed to withdraw from the study. It will take approximately 40 minutes to completion of the self-reported questionnaire. A teacher and team member were always present in the classroom but was not permitted to intervene in the research procedure. The same investigation carried out after six months. Approval for the design and data collection procedures was obtained by the Department of Psychological Education of Elementary and Secondary Schools of the Guangdong Province Administration.

Study participants were randomly selected from among the total student population attending high school within the region and were registered with the Guangzhou secondary school registry. A stratified random sampling method (with stratification according to the proportion of students in metropolitan and rural areas) was used for sample generation. At baseline, 1618 high school students provided usable information. Data were collected via health survey. Anthropometric indices and lipid profiles were evaluated within 1 week of survey administration. The cohort was then followed up for 6 months, with the survey conducted again to evaluate mental health outcomes at the end of follow-up. For the present study, a “community-based sample” cohort was generated from the larger cohort by screening for anxiety and depression at baseline. After data merging and cleaning, 1348 study participants, aged 12–18 yr, with an average age of 15.34 (± 1.81) yr, completed both pre-test and post-test phases of the study.

### Measures

Health survey information was collected using a self-administered questionnaire, which investigated demographics, family-based factors, and lifestyle factors. Questions pertained to age, gender, grade level, number of siblings, place of residence, perceptions of familial financial situation, parental education, time spent with parents during elementary school, skipping breakfast, sleeping hours, and physical activity.

Anthropometric measurements were performed by experienced technicians in a quiet room. Each participant’s height and weight was measured with lightly clothed and barefoot using an automatic instrument (KN-5000A, Nakamura, and Tokyo Japan). Body mass index (BMI) was calculated as weight divided by height squared (kg/m^2^). Participants were classified as overweight/obese or non-overweight, based on baseline measurements of weight. Overweight and obesity categories were defined according to the Working Group on Obesity in China (WGOC) BMI cutoff points based on age- and sex-specific screening criteria[19]. In this study, average BMI in the overweight/obesity group was 25.12 ± 2.76 kg/m^2^. Average BMI in the non-overweight group was 18.48 ± 2.04 kg/m^2^. This difference was statistically significant (*t* = 35.26, *P* = 0.001).

### Mental health

Anxiety was measured using the Zung Self-rating Anxiety Scale [20] and depression was assessed using the Zung Self-rating Depression Scale [21] at baseline as well as at follow-up. The Self-rating Anxiety Scale (SAS) and the Self-rating Depression Scale (SDS) are 20-item scales that evaluate clinical symptoms of anxiety/depression in adults and adolescents. Questions are designed to evaluate the severity of anxiety/depression by asking patients how often they have experienced certain conditions or were in certain states of mind within the 3 months leading up to administration of the survey. Response scores to each item ranged from 1 to 4, so total scores ranged from 20 to 80 points. SAS scores were further categorized into 4 levels of anxiety severity: normal, less than 45; mild to moderate, 45 to 59; marked to severe, 60–74; and extreme, 75 or greater, according to the recommended cutoff [20]. SDS scores were also further categorized into 4 levels: normal, less than 50; mild depression, 50 to 59; moderate to marked major depression, 60 to 69; and severe or extreme major depression, ≥ 70, according to the recommended cutoff [21]. The outcome measure was further dichotomized into normal, less than 50, and depressed, 50 or greater, for ease of analysis. The Chinese versions of both instruments were validated in a Chinese adolescent population with good validity and reliability [22, 23].

### Statistical analysis

Data were analyzed using IBM SPSS Statistics 19.0 for Windows. The numerical variables were expressed by the means of the calculation of means and standard deviations. First, Chi-squared or the Wilcoxon rank-sum test was conducted to examine differences, such a number of siblings, and all variables of interest between the overweight/obesity group and the non-overweight group. Logistic regression analysis was performed to identify potential risk factors for overweight/obesity, including number of siblings. Potential confounding variables were entered in the model based on the significance level of these variables in bivariate analysis. Variables with *P* ≤ 0.05 were considered as potential confounders in subsequent analysis for the adjusted relationship between exposure and outcome variables. Finally, on the basis of the existing two groups: overweight/obesity group and non-overweight group, logistic regression predicating the disability outcome measures (i.e., symptoms of depression and anxiety) at 6-month follow-up was used for multivariate analysis. The same analyses as described previously were carried out.

## Results

Among the 1348 participants, 9.2% of boys (n = 53) and 5.9% of girls (n = 45) being only children and respectively 1.9% (n = 11) and 3.3% (n = 25) belonging to non-only children were overweight/obesity. To better assess the effects of obesity, we performed Student’s *t*-test to compare other anthropometric indices and lipid profile between only children and children with siblings (S1 Text). Anthropometric parameters such as waist-hip ratio (WHR), triglyceride level (TG), and total cholesterol (TC) also differed significantly between only children and those living with siblings (S1 Text).

**Fig 1:**
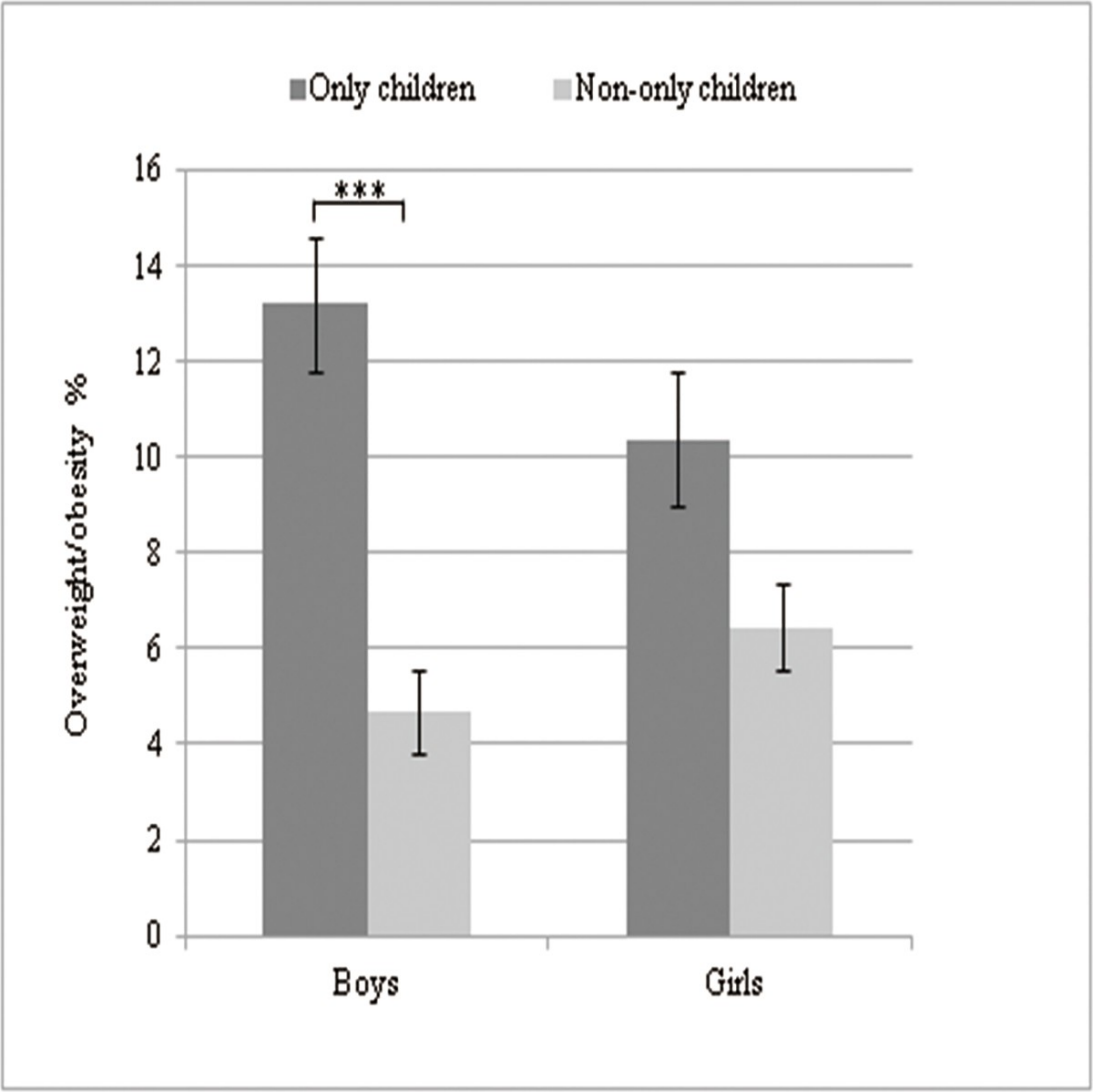
Gender-specific prevalence of overweight/obesity among boys (n = 579) and girls (n = 769), stratified by existence of siblings.

**Fig 2:**
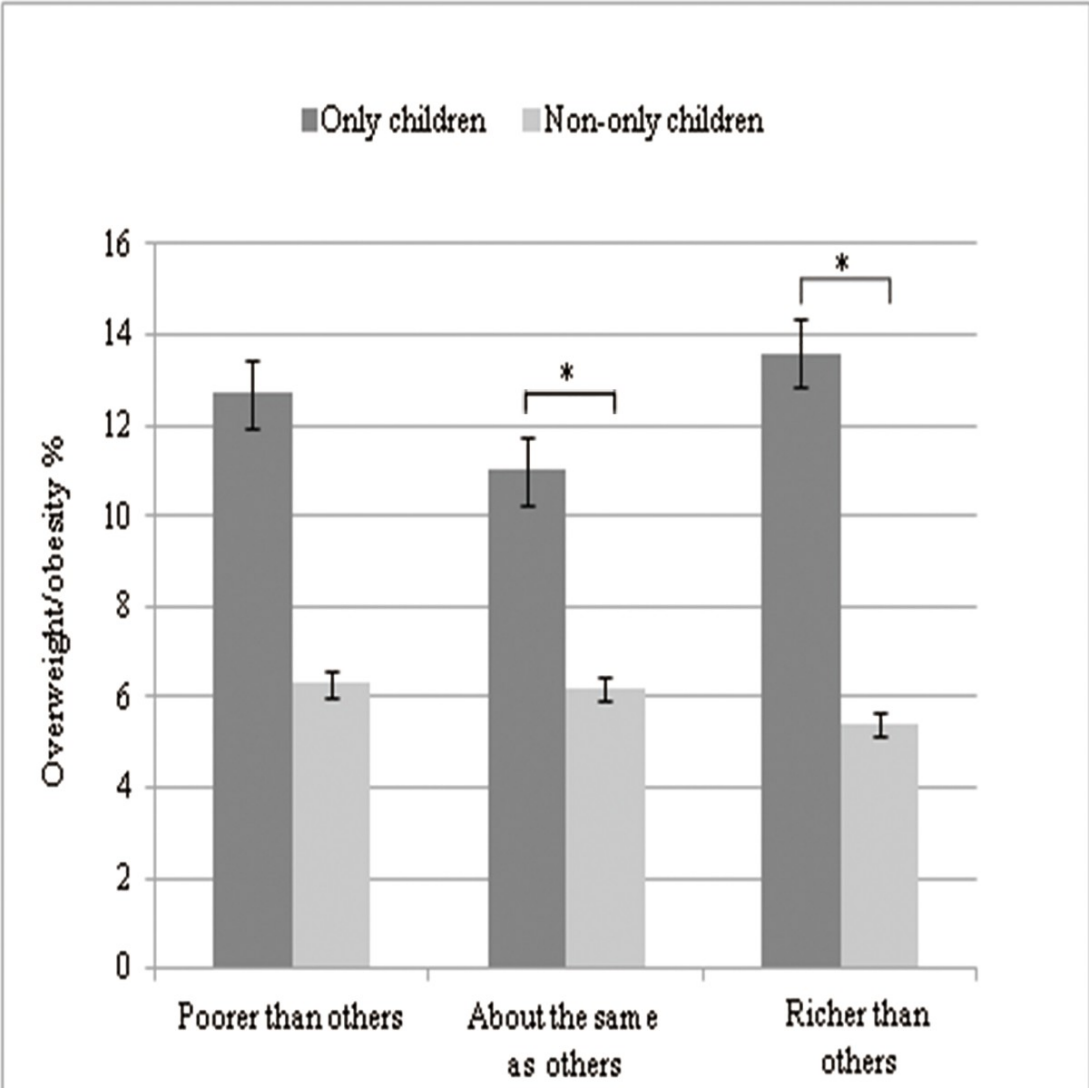
Association between familial socioeconomic status and prevalence of overweight/obesity among groups with low (n = 123), mid-level (n = 786) and high socio-economic status (n = 313), stratified by existence of siblings.

Table 1 presents family-based and lifestyle factors for all study participants. The overweight/obesity rate of only children was 11.8%, higher than that of having siblings (6.0%). The difference had statistical significance (*χ*^2^ = 13.21 *P*<0.001). In addition, overweight/obesity was significantly higher among adolescents having no siblings in boys (*P*<0.001) (Fig 1). Adolescents whose families resided in the city (10.3%) were significantly more likely to be overweight/obese (*P* = 0.01) than were those living in semirural (9.2%) or rural areas (1.7%). No association between perceived family financial situation and overweight/obesity was observed. After stratification for family financial situation, higher prevalence of overweight/obesity was observed among families with higher socioeconomic status, for both only children as well as children with siblings (Fig 2).

**Table 1:** Characteristics of the non-overweight and overweight/obesity groups.

Variables	Non-overweight N = 1222		Overweight/Obesity N = 126		Significance	
	n	%	n	%	Χ^2^	P value
**Family-based factors**						
*Number of siblings*						
None (only child)	687	56.20%	92	73.00%	13.21	0.000
One or more	535	43.80%	34	27.00%		
*Family location*					9.22	0.01
Rural	116	9.50%	2	1.60%		
Semirural	227	18.60%	23	18.30%		
Urban	879	71.90%	101	80.20%		
*Family financial situation*					0.29	0.87
Poorer than others	123	10.10%	12	9.50%		
About the same as others	786	64.30%	79	26.70%		
Richer than others	313	25.60%	35	27.80%		
*Father’s education level*					6.72	0.035
Less than senior high school	102	8.30%	5	4.00%		
Senior high or technical school	945	77.30%	94	74.60%		
University or higher	178	14.40%	27	21.40%		
*Mother’s education level*					2.43	0.291
Less than senior high school	120	9.80%	9	7.10%		
Senior high or technical school	966	79.10%	98	77.80%		
University or higher	136	11.10%	19	15.10%		
*How much time do you spend with your father in elementary school*?					7.45	0.006
Less than half	331	27.10%	20	15.90%		
More than half	891	72.90%	106	84.10%		
*How much time do you spend with your mother in elementary school*?					8.29	0.004
Less than half	155	12.70%	5	4.00%		
More than half	1067	87.30%	121	96.00%		
**Lifestyle factors**						
*Skipping breakfast*					4.54	0.033
Yes	143	11.70%	23	18.30%		
No	1079	88.30%	103	81.70%		
*Sleeping hours*					2.16	0.34
<6.0	105	8.60%	10	7.90%		
6.0–8.0	812	66.40%	77	61.10%		
8.0+	305	25.00%	39	31.00%		
*Vigorous physical activity*					2.87	0.075
Yes	277	22.70%	37	29.40%		
No	945	77.30%	89	70.60%		
*Moderate physical activity*					3.78	0.052
Yes	157	12.80%	24	19.00%		
No	1065	87.20%	102	81.00%		

Interestingly, as the father's level of education improved, the rate of overweight/obesity increased (*χ*^2^ = 6.72 *P*<0.05), from 4.7% for children of fathers who had left school before senior high school, to 9.0% of children with fathers who had completed senior high or technical school, to 13.4% of children whose fathers had completed college or an advanced degree. However, mother’s education level was not associated with overweight/obesity. Students in elementary school who spent more than half their time with parents were frequently overweight/obese(*P*_*s*_<0.01). In terms of lifestyle factors, students who skipped breakfast were also frequently overweight/obese. A marginally significant difference between the overweight and non-overweight groups was observed regarding vigorous and moderate physical activities. Sleeping hours were not associated with overweight and obesity.

Crude and adjusted ORs of number of siblings for overweight/obesity were calculated in Table 2. The result showed that number of siblings was independently associated with higher risk of overweight/obesity. With univariate analysis, the logistic regression results showed that being an only child (OR: 2.17, 95% CI: 1.46–3.22) significantly increased the OR for overweight/obesity compared with those having one or more siblings. After age, family location, father’s education level, parent-child relationship in elementary school, and skipping breakfast were adjusted, only children as compared to more than one sibling (aOR 1.68, 95% CI 1.06–2.65) who were used as a reference variable significantly increased the OR for being overweight/obesity.

**Table 2:** Logistic regression models examining the association between the number of siblings and overweight/obesity.

Variables	Total N	Overweight/Obesity n(%)	Crude			Adjusted		
			OR	95%CI	*P* value	OR^a^	95%CI	*P* value
*Number of siblings*								
Only child	687	92(11.81%)	2.17	1.46–3.22	0.000	1.68	1.04–2.51	0.034
One or more	535	34(5.98%)	1(ref)			1(ref)		

Abbreviations: CI, confidence interval; OR, odds ratio.

^a^ Adjusted for age, family location, father’s education level, parent-child relationship in elementary school, and skipping breakfast.

After stratifying study participants based on weight status, we calculated adjusted ORs for number of siblings and depression and anxiety at the end of the 6-month follow-up period in Tables 3 and 4. Only children who were overweight and obese resulted in a lower OR for depression (aOR, 0.19; 95% CI, 0.34–0.86, *P* for trend<0.05). However, there was no significant association between number of siblings and depression in the non-overweight group. For all non-overweight, overweight, and obese individuals, no significant relationship between number of siblings and anxiety was observed at follow-up.

**Table 3:** Adjusted odd ratios of anxiety and depression at follow-up for the number of siblings in overweight/obesity group.

Overweight/Obesity										
	Total N	*Depression*				Total N	*Anxiety*			
		n(%)	aOR^a^	95%CI	P value		n(%)	aOR^a^	95%CI	P value
*Number of siblings*										
Only child	87	3(3.4%)	0.19	0.34–0.86	0.031	88	2(2.3%)			
One or more	32	5(15.6%)	1(ref)			33	0	1(ref)		

Abbreviations: CI, confidence interval; aOR, adjusted odds ratio.

^a^ Adjusted for age, sex, family location, and family financial situation

**Table 4:** Adjusted odd ratios of anxiety and depression at follow-up for the number of siblings in non-overweight group.

Non-overweight										
	Total N	*Depression*				Total N	*Anxiety*			
		n(%)	aOR^a^	95%CI	P value		n(%)	aOR^a^	95%CI	P value
*Number of siblings*										
Only child	657	56(8.5%)	1.13	0.71–1.81	0.612	649	22(3.4%)	1.5	0.78–2.91	0.23
One or more	501	40(8.0%)	1(ref)			501	18(3.6%)	1(ref)		

Abbreviations: CI, confidence interval; aOR, adjusted odds ratio.

^a^ Adjusted for age, sex, family location, and family financial situation

## Discussion

The aims of this study were to investigate effects of sib-size and obesity status on adolescent mental health, including anxiety and depression, using a community-based sample in Southeast China. These relationships were evaluated over a 6-mo period. The present results support our main hypothesis suggesting that only children had significantly higher odds of obesity in young adulthood, compared with children with siblings. However, after adjusting for potential confounding factors, only children who were overweight and/or obese had a lower OR for depression at the 6-mo follow-up. No such relationship was demonstrated for non-overweight individuals. This study is the first to demonstrate that mental health sequelae associated with obesity differ between only children and those living with siblings.

### Obesity among only children

The present study found that the overweight/obesity rate of only children was 11.8%, significantly higher than that of having siblings (6.0%). Only children also had higher odds of obesity than adolescents with siblings. This result was consistent with the previous studies. The inverse relationship was generally found between number of siblings and the prevalence of overweight/obesity [13, 16, 17, 24]. One possible explanation for this association between sib-size and obesity is that sibling(s) might also serve as a stimulus for child-to-child interactions, cooperative play, or activities that increase the time each child devotes to physical activity [25]. In addition, similar results were found in other anthropometric indices for identifying dyslipidemia with obesity between only children and adolescents with siblings, including waist circumference (WC), waist-to-height ratio (WtHR), waist-hip ratio (WHR) and total cholesterol (TC), fasting blood glucose (FBG). Analyses were stratified by sex. The results showed significant differences between groups. This result was mainly driven by males, in contrast to previous reports of a negative correlation between number of siblings and both fat mass and fat-free mass in women [26]. One possible explanation for this pattern stems for the preference for male children in China. Rather, an only daughter in a family may not be as strongly desired and thus is more likely to be treated the same whether or not she is an only child.

Furthermore, the existence of more siblings leads to an increase in family size, which has an inverse effect on family income and, consequently, on obesity. A study conducted in Brazil demonstrated a direct association between income and obesity in adolescents of both sexes [7]. We also observed that only children identified as being overweight/obese reported higher prevalence of parents with higher education, living in an urban area, and perception of high economic status. Only children from families having higher socioeconomic class tend to lead an unhealthy life. For example, previous studies showed that only children had significantly higher intake of many nutrients and nutrients/1000 kcal [26]. Compared with children who had siblings, only children spent more time each day engaged in low-intensity physical activity [27] and spent less time each day engaged in physical activity of moderate-to-vigorous intensity [27, 28]. A previous study in the United States also showed that, compared with adolescents living in families with two or more siblings, only children were more likely to have a television in the adolescents' bedroom, spend over an hour in front of screen per day, and eat a meal infrequently with all the family members living in the household [29]. Therefore, behavioral modification has been recommended has been suggested as the best approach to prevent and manage obese [30].

### Imbalance in obesity and mental health among only children

The results presented above indicate that only children who were initially free of mental health problems but who were overweight/obesity had lower risk for depression, which runs counter to our hypothesis that only children with obesity would also have a higher risk for anxiety and depression at the end of the 6-mo follow-up period. This implies that non-only-children with overweight and obesity, additional sibling(s) may be an adverse situation that is more likely to result in adolescents’ ongoing mental health problems. This is surprising; given previous studies found that only children, especially, have variously been depicted as disadvantaged as a result of “sibling deprivation,” which may lead to poorer mental health outcomes including lower subjective well-being, more pessimistic, more depressed and less happy [10, 18].

One possible explanation for this discrepancy is that children who come from a larger family experience negative social consequences due to the social stigma around having more than one child in China. Only children are portrayed as good, whereas children with siblings are portrayed as bad. It seems like that these children, rather than only children, have higher risk for poor adjustment and subsequent psychopathology [15]. Status as an only child seems to confer a marginal protective effect: only children were found to be consistently better off regarding social adjustment, popularity, and willingness to confide in others. Only children were also less likely to be victims of bullying. This suggests that in the absence of siblings they rely more on friends for social interaction and support. In addition, adolescents with overweight and obesity may experience stigmatization, poor body image and low self-esteem increasing their vulnerability to depression [31, 32], implying that obese can be a higher risk factor for adolescent depression. Therefore, suffering from obese, only children may have more support to cope with these problems than those having siblings.

Another potential explanation for the observed results is that the presence of siblings in a family may indirectly affect the adolescent’s mental health outcomes through effects on the behavior of parents. According to the resource dilution theory [33], a larger number of children may not only dilute a family’s substantial resources (financial and physical), but also parental practices such as attention, intervention, caretaking, etc. Therefore, parents of only children may not only invest more in their only child [34], but also be more responsive to the child’s needs; this may lead to a greater sense of security, intellectual competence, self-esteem, and psychological confidence of the child [35]. Parents of only children may also be more able to interact with their children in ways that promote desirable development. Thereafter, without suffering from resource dilution, only children could possess a higher level of mental health.

The OCP has lasted more than 30 years and has significantly controlled population growth [9]. The younger generation of Chinese individuals includes many who are only children. However, aging of the population and low fertility have led to decreased labor supply, which suggests that China’s population policy requires adjustment. Although the Universal Two-child Policy was officially implemented in January 1, 2016, some extreme reactions of only children in a family were shocking [36, 37]. It was reported that a 13-year-old junior high school student whose parents wanted to give birth to a second child, kept playing truant, ran away from home, and even threatened their parents with death, which lead to the elderly pregnant mother terminating pregnancy in the hospital ultimately [36].

### Strengths and limitations

The primary strength of the present study is its nature as a cohort study. The results provide further information that adolescents with overweight and obesity, additional sibling(s) is a possible risk factor for the development of mental health, particularly depression. This finding seems to demonstrate a relationship between the existence of siblings and symptoms of depression among adolescents. However, the study has certain limitations. First, not all potential confounders were measured and adjusted in the analysis. As overweight and obesity are complex phenomena, the main causes of overweight and obesity are multifactorial and include genetic variation, history of familial obesity, birth weight, and social and environmental determinants (e.g., intake of energy-dense food, energy expenditure), which were not assessed in this study. An additional limitation of the study is that six months of follow-up is not sufficient time to assess the prevalence of overweight and obesity in the context of maturation, which is the approach recommended by the World Health Organization [38].

## Conclusion

The current study suggests an effect of obesity on mental health among only children. Being an only child was associated with overweight and obesity among adolescents and negatively associated with symptoms of depression at follow-up. These findings support the need to design evidence-based adolescent health policy and to implement targeted interventions to address adolescent adiposity and mental health problems, considering the impact of family-based factors.

### Ethics approval and consent to participate

Institutional ethics approval for the study was granted by the Department of Psychological Education of Elementary and Secondary Schools of the Guangdong Province Administration. Parents did not provide written consent, but students younger than 16 yr were instructed to obtain verbal consent from parents before filling in the self-reporting questionnaire. For students > 16 yr (age of self-consent), consent was implicated by a voluntary response to the questionnaire. In addition, students with severe depression or anxiety in pre-test and post-test were referred for appropriate care. The institutional review board approved this consent procedure.

## Supporting information

S1 Text Comparison of anthropometric indices and lipid profile between only children and non-only children, by gender and overall. (DOCX)

S2 Text Dataset of imbalance in obesity and mental health among “Little Emperors” in China. (XLSX)
