# Supplementary material for: Imbalance in obesity and mental health among “little emperors” in China
Source: PLoS One. 2019 Apr 10;14(4):e0207129. doi: 10.1371/journal.pone.0207129 (PMC6457487; doi:10.1371/journal.pone.0207129)
Supplement: S1 Text — (DOCX) [file pone.0207129.s001.docx]

**Comparison of only-children and non-only children in terms of anthropometry and lipid profile**

In the contexts of clinical practice and biomedical research, body mass index (BMI) is often used to identify overweight or obese adolescents. Although BMI remains the most popular tool for detecting obesity, its main limitation is its inability to consider body fat distribution. To better assess the evolution of obesity, we performed Student’s *t*-test to compare anthropometric parameters associated with lipid disorders between only children and children with siblings.

**Material and Method**

**Participants and Procedure**

Study participants were high school students from urban and rural areas in Guangzhou city, Guangdong Province, Southeast China. The study included 1348 middle schools in 1 urban region and 2 rural regions.

**Laboratory Analysis**

Venous blood samples were collected from adolescents after ≥8 hr of fasting. Serum was separated by centrifugation at 1500×g for 15 min at 4°C within 2 hr and stored at −80°C until testing. Serum levels of triglycerides (TG), total cholesterol (TC), fasting blood-glucose (FBG), low-density lipoprotein cholesterol (LDL-C), high-density lipoprotein cholesterol (HDL-C), total bilirubin, and direct bilirubin were measured using commercial colorimetric kits (Biosino Biotechnology Company Ltd, Beijing, China) and an automated analyzer (Hitachi 7150, Tokyo, Japan). Inter-and intra-assay coefficients of variation for all measured lipid parameters were <5%.

**Anthropometric Measurements**

Anthropometric measurements, including hip circumference (HC), waist circumference (WC), and blood pressure, were measured by experienced technicians. Body weight and height were measured by automatic instruments (KN-5000A, Nakamura, Tokyo, Japan). WC and HC were measured to the nearest 0.1 cm. Systolic and diastolic blood pressure measurements were obtained using a sphygmomanometer (Yutu XJ1ID, Shanghai, PRC) after the study participant had remained in a seated position for 5 min.

**Statistical analysis**

Data were analyzed using SPSS 19.0 software. Measurement data are expressed as mean ± standard deviation. Enumeration data are expressed as a percentage of total case number. Student’s *t*-test was used to compare anthropometric indices and lipid profile between only children and children with siblings. Differences with *P*<0.05 were considered statistically significant.

**Results**

**Comparison of Only Children with Children with Siblings**

Among 1348 participants, 9.2% of boys (n=53) and 5.9% of girls (n=45), respectively, were only children. Among only children, 1.9% (n=11) were overweight; among non-only children, 3.3% (n=25) were overweight. Student’s *t*-test was used to compare anthropometric indices and lipid profile between only children and children with siblings. Only children presented higher values of anthropometry than did those living with siblings (*P*<0.05) for all measures except WC, for which mean was lower among only children, compared with those living with siblings (*P<*0.05). In terms of lipid profile, only TC and FBG differed significantly between groups. These results suggest that anthropometric parameters differ significantly between only children and those with siblings. When stratified by sex, the results showed significant differences among groups, but this result was mainly driven by only children who were male.

| **Table S1:** Evolution the anthropometry and lipid profile between the only-children and non-only children by gender and overall. | | | | | | | | |
| --- | --- | --- | --- | --- | --- | --- | --- | --- |
|  | *Boys(n=579)* | |  | *Girls(n=769)* | |  | *Total(n=1348)* | |
|  | *Only-child* | *Non-only child* |  | *Only-child* | *Non-only child* |  | *Only-child* | *Non-only child* |
| ***Anthropometry*** |  |  |  |  |  |  |  |  |
| Body Mass Index (BMI) (kg/m2) | 19.51±3.37^a^ | 18.31±2.41 |  | 19.14±2.77 | 19.15±2.52 |  | 19.31±3.08^a^ | 18.84±2.51 |
| Hip Circumference (HC)(cm) | 84.03±7.19^a^ | 81.68±6.36 |  | 83.23±6.10 | 83.55±5.62 |  | 83.61±6.65^a^ | 82.86±5.97 |
| Waist Circumference (WC) (cm) | 67.74±9.02^a^ | 65.07±6.97 |  | 67.57±8.50^a^ | 70.48±6.69 |  | 69.73±7.68^a^ | 67.65±8.75 |
| Waist-Height Ratio(WtHR) | 0.42±0.05^a^ | 0.39±0.04 |  | 0.43±0.05^a^ | 0.46±0.04 |  | 0.44±0.05^a^ | 0.42±0.05 |
| Waist-Hip Ratio (WHR) | 0.87±0.06^a^ | 0.81±0.07 |  | 0.80±0.06 | 0.79±0.05 |  | 0.84±0.07^a^ | 0.81±0.06 |
| Systolic Blood Pressure(SBP) (mmHg) | 108.73±8.78 | 108.5±8.79 |  | 106.58±8.27 | 106.41±8.34 |  | 107.62±8.58 | 107.19±8.56 |
| Diastolic Blood Pressure(DBP) (mmHg) | 66.06±7.36^a^ | 68.35±6.68 |  | 64.63±5.88^a^ | 66.90±6.12 |  | 65.32±6.67^a^ | 67.44±6.37 |
| ***Lipid profile*** |  |  |  |  |  |  |  |  |
| Triglyceride (TG) (mmol/L) | 1.16±0.53^a^ | 0.93±0.39 |  | 1.02±0.42 | 1.00±0.47 |  | 1.08±0.48 | 0.97±0.44 |
| Total Cholesterol (TC) (mmol/L) | 4.22±0.73^a^ | 3.91±0.66 |  | 4.17±0.69 | 4.12±0.74 |  | 4.16±0.71^a^ | 4.09±0.73 |
| Fasting Blood Glucose(FBG)(mmol/L) | 4.69±0.45^a^ | 4.58±0.41 |  | 4.62±0.38 | 4.54±0.42 |  | 4.65±0.42^a^ | 4.57±0.41 |
| LDL Cholesterol(LDL-C) (mmol/L) | 2.66±0.69^a^ | 2.36±0.64 |  | 2.55±0.61 | 2.61±0.66 |  | 2.53±6.65 | 2.51±0.66 |
| HDL Cholesterol(HDL-C) (mmol/L) | 1.33±0.25 | 1.38±0.29 |  | 1.41±0.27 | 1.39±0.29 |  | 1.37±0.26 | 1.39±0.29 |

Abbreviation: ^a^ Significant increase/decrease between only children and adolescent with siblings (*P≤*0.05).
